# High Stomatal Conductance in the Tomato *Flacca* Mutant Allows for Faster Photosynthetic Induction

**DOI:** 10.3389/fpls.2020.01317

**Published:** 2020-08-25

**Authors:** Elias Kaiser, Alejandro Morales, Jeremy Harbinson, Ep Heuvelink, Leo F. M. Marcelis

**Affiliations:** ^1^Horticulture and Product Physiology, Department of Plant Sciences, Wageningen University, Wageningen, Netherlands; ^2^Centre for Crop Systems Analysis, Department of Plant Sciences, Wageningen University, Wageningen, Netherlands; ^3^Molecular Plant Physiology, Institute of Environmental Biology, Utrecht University, Utrecht, Netherlands; ^4^Plant Ecophysiology, Institute of Environmental Biology, Utrecht University, Utrecht, Netherlands

**Keywords:** abscisic acid, air humidity, CO_2_ concentration, fluctuating irradiance, dynamic photosynthesis, stomatal conductance

## Abstract

Due to their slow movement and closure upon shade, partially closed stomata can be a substantial limitation to photosynthesis in variable light intensities. The abscisic acid deficient *flacca* mutant in tomato (*Solanum lycopersicum*) displays very high stomatal conductance (*g_s_*). We aimed to determine to what extent this substantially increased *g_s_* affects the rate of photosynthetic induction. Steady-state and dynamic photosynthesis characteristics were measured in *flacca* and wildtype leaves, by the use of simultaneous gas exchange and chlorophyll fluorometry. The steady-state response of photosynthesis to CO_2_, maximum quantum efficiency of photosystem II photochemistry (*F_v_/F_m_*), as well as mesophyll conductance to CO_2_ diffusion were not significantly different between genotypes, suggesting similar photosynthetic biochemistry, photoprotective capacity, and internal CO_2_ permeability. When leaves adapted to shade (50 µmol m^−2^ s^−1^) at 400 µbar CO_2_ partial pressure and high humidity (7 mbar leaf-to-air vapour pressure deficit, VPD) were exposed to high irradiance (1500 µmol m^−2^ s^−1^), photosynthetic induction was faster in *flacca* compared to wildtype leaves, and this was attributable to high initial *g_s_* in *flacca* (~0.6 mol m^−2^ s^−1^): in *flacca*, the times to reach 50 (*t_50_*) and 90% (*t_90_*) of full photosynthetic induction were 91 and 46% of wildtype values, respectively. Low humidity (15 mbar VPD) reduced *g_s_* and slowed down photosynthetic induction in the wildtype, while no change was observed in *flacca*; under low humidity, *t_50_* was 63% and *t_90_* was 36% of wildtype levels in *flacca*. Photosynthetic induction in low CO_2_ partial pressure (200 µbar) increased *g_s_* in the wildtype (but not in *flacca*), and revealed no differences in the rate of photosynthetic induction between genotypes. Effects of higher *g_s_* in *flacca* were also visible in transients of photosystem II operating efficiency and non-photochemical quenching. Our results show that at ambient CO_2_ partial pressure, wildtype *g_s_* is a substantial limitation to the rate of photosynthetic induction, which *flacca* overcomes by keeping its stomata open at all times, and it does so at the cost of reduced water use efficiency.

## Introduction

In the leaves of higher plants, stomata balance carbon uptake against water loss. They achieve this balance by dynamically regulating stomatal aperture in response to intrinsic and extrinsic factors. Typically, stomatal aperture decreases in low irradiance or darkness, and increases in high irradiance. Stomatal opening after sudden increases in irradiance is slow compared to changes in Calvin cycle metabolism (McAusland et al., 2016), with time constants in the range of 4 to 29 min (Vico et al., 2011). Due to the slow opening of stomata, the increase of stomatal conductance (*g_s_*, mol m^−2^ s^−1^) from a low initial value is assumed to be one of the three main limitations of net photosynthesis rate (*A*, µmol m^−2^ s^−1^) in response to increases in irradiance (e.g., Urban et al., 2008; Kaiser et al., 2016; Li et al., 2016; Kaiser et al., 2017a; Zhang et al., 2018; Zhang et al., 2020). Given that solar irradiance incident on a leaf often fluctuates, these dynamic limitations of photosynthesis decrease photosynthetic irradiance use efficiency (Morales et al., 2018). Improving *g_s_*, including its dynamics, is an attractive means with which to improve both irradiance and water use efficiency (Lawson and Blatt, 2014; Vialet-chabrand et al., 2017). Increases in the limitation imposed upon *A* by *g_s_* can be identified via transient decreases of leaf internal CO_2_ partial pressure (*C_i_*, µbar). The other two main limitations during photosynthetic induction arise from slow rates of change in the activity of enzymes involved in ribulose-1,5-bisphosphate (RuBP) regeneration, and from slow activation of Rubisco (Pearcy et al., 1996; Way and Pearcy, 2012; Kaiser et al., 2015; Kaiser et al., 2018). These limitations occur additionally to the limitations at steady state due to, e.g., the rate of electron transport, Calvin cycle metabolism, sucrose metabolism, or mesophyll conductance (*g_m_*, mol m^−2^ s^−1^).

Disentangling stomatal and other limitations during photosynthetic induction is difficult. Many treatments affecting *g_s_* also affect other transient limitations of photosynthetic induction, such as leaf temperature (Kaiser et al., 2017a) and salt stress (Zhang et al., 2018). Similarly, some genetic mutations affecting *g_s_*, such as the abscisic acid (ABA) deficient *aba2-1* mutant in *Arabidopsis thaliana*, showed enhanced *A*/*C_i_* responses compared to its wildtype, Col-0 (Kaiser et al., 2016), suggesting pleiotropic effects, which may confound effects of altered *g_s_*. Also, models estimating transient stomatal limitation have often been based on linear – not curvilinear – *A*/*C_i_* relationships (Woodrow and Mott, 1989; Tinoco-Ojanguren and Pearcy, 1993), or are based on steady-state *A*/*C_i_* responses (Kaiser et al., 2017a). In fact, the maximum rate of carboxylation (*V_cmax_*), for example, increases strongly during photosynthetic induction (Soleh et al., 2016; Taylor and Long, 2017). In another approach, the limitation during induction was attributed to *g_s_* alone up to the point where *A* reached 95% of the steady-state value (McAusland et al., 2016), entirely ignoring limitations by RuBP regeneration and Rubisco activation kinetics. Tools to better separate stomatal from other limitations are thus warranted, and mutants or transformants with substantially altered stomatal characteristics but similar photosynthetic biochemistry (Raissig et al., 2017; Papanatsiou et al., 2019; Tomimatsu et al., 2019; Kimura et al., 2020; Yamori et al., 2020) can be counted among such tools.

The tomato (*Solanum lycopersicum* L.) *flacca* mutant has a 80% to 90% lower ABA content than its wildtype (Tal and Nevo, 1973; Sagi et al., 2002). *Flacca* leaves exhibit a very high *g_s_*, without affecting the *A*/*C_i_* response, suggesting that photosynthetic capacity is independent of ABA (Bradford et al., 1983). Lack of ABA has not been found to affect *g_m_* in the *aba-1* mutant of *Nicotiana plumbaginifolia* (Mizokami et al., 2015) but to our knowledge has not been determined in *flacca*. The aim of this study is to determine the dynamic limitations of photosynthetic induction due to *g_s_* in tomato leaves. For this, steady-state and dynamic photosynthesis characteristics were measured in the ABA-deficient *flacca* mutant and its wildtype, by the use of simultaneous gas exchange and chlorophyll fluorometry.

## Materials and Methods

### Plant Material

Seeds of tomato cv. Rheinlands Ruhm wildtype (LA0535) and *flacca* (LA0673) were obtained from the Tomato Genetics Resource Center (University of California, Davis, USA). Seeds were germinated in stonewool plugs (Grodan, Roermond, NL). A week after sowing, they were transferred to stonewool cubes (10 cm × 10 cm × 7 cm; Grodan). Plants were grown in a climate chamber under a day/night cycle of 16/8 h (day/night), 20/18°C temperature, ambient CO_2_ partial pressure, 70% relative air humidity, and 154 µmol m^−2^ s^−1^ photosynthetically active radiation (PAR; measured 10 cm above table height), which was provided by fluorescent tubes (Master TL-D 58W/840 Reflex Eco; Philips, Eindhoven, the Netherlands). Stonewool cubes were standing in a layer (height, 1–2 cm) of nutrient solution (Yara Benelux B.V., Vlaardingen, the Netherlands), which was replenished every 1 to 2 days and contained 12.4 mM NO_3_^−^, 7.2 mM K^+^, 4.1 mM Ca^2+^, 3.3 mM SO_4_^2−^, 1.8 mM Mg^2+^, 1.2 mM NH_4_^+^, 1.1 mM PO_4_^3−^, 30 μM BO_3_^3−^, 25 μM Fe^3+^, 10 μM Mn^2+^, 5 μM Zn^2+^, 0.75 μM Cu^+^, and 0.5 μM MoO_4_^2−^ (EC 2.1 dS m^−1^, pH 5.5). Between 1 and 4 weeks after sowing, *flacca* plants were sprayed daily with a solution containing 10 μM ABA, 0.01% (w/v) Triton-X, and 0.1% (v/v) ethanol (Bradford et al., 1983), using commercially available horticultural hand sprayers. Wildtype plants were sprayed with a control solution containing 0.01% Triton-X and 0.1% ethanol. Untreated *flacca* plants are smaller and show much higher transpiration rates than the wildtype, together with leaf epinasty and strong root formation along the stem (Tal, 1966). Growing *flacca* with application of ABA causes plants to grow similarly well as the wildtype (Imber and Tal, 1970). When the application of ABA is stopped, *flacca* reverts to its mutant phenotype within days, including always-open stomata (Imber and Tal, 1970). All chemicals were purchased from Sigma (St. Louis, MO, USA).

### Measurements

When plants were between 5 and 6 weeks old, the fourth leaf, counting from the bottom, was selected for measurements. ABA spraying was stopped seven days before the start of measurements, to allow the high *g_s_* phenotype of *flacca* to reassert itself. Measurements were performed in a lab, using the LI-6400 XT photosynthesis system (LI-COR Biosciences, Lincoln, Nebraska, USA) equipped with a fluorescence chamber (leaf area: 2 cm^2^). Conditions inside the leaf chamber during measurements were: 25°C chamber temperature, 7 mbar leaf-to-air vapour pressure deficit (VPD; except when stated otherwise) and a flow rate of 500 µmol s^−1^. Irradiance was provided by LEDs in a 90/10 red/blue irradiance mixture, with peak intensities at wavelengths of 635 and 465 nm, respectively. For all measurements, five plants per genotype were used (n = 5).

All measurements were performed on the same spot of a leaf, to reduce measurement noise caused by spatial variation (Lawson and Weyers, 1999; Matthews et al., 2017): (a) dark-adapted *F_v_/F_m_*, (b) *A*/PAR curves at 2% oxygen, (c) *A*/*C_i_* curves at 2% oxygen, (d) *A*/*C_i_* curves at 21% oxygen, (e-g) photosynthetic induction under three different environmental conditions (described below). While measurements a-d were performed in the same sequence, the order of photosynthetic induction measurements was randomized for each plant. Values of *A* were corrected for CO_2_ leakage based on the manufacturers' suggestions. Measurements were started at 7:30 in the morning and took 8 to 9 h to complete per leaf.

### Dark-Adapted F_v_/F_m_ and Net CO_2_ Exchange in Darkness

Leaves were dark-adapted for 20 minutes. Then, net CO_2_ exchange in darkness (*A*_dark_) was logged, after which a weak measuring beam was turned on to measure *F_o_*. Then, *F_m_* was determined by exposing the leaf to a single-pulse saturating flash of ~9.000 µmol m^−2^ s^−1^ intensity and 1-s duration. Dark-adapted *F_v_/F_m_* was calculated as *F*_v_/*F*_m_ *= (F*_m_ − *F*_o_)/*F*_m_.

### A/PAR Curves at 2% Oxygen

A gas mixture containing 2% oxygen and 98% nitrogen was fed to the inlet of the LI-6400 XT. Leaf external CO_2_ partial pressure (C_a_) was set to 2000 µbar, and irradiance was set to 200 µmol m^−2^ s^−1^. After reaching steady-state *A*, irradiance was decreased in steps of 150, 100, 70, 50, and 30 µmol m^−2^ s^−1^, and *A* was logged for 30 s after reaching steady-state *A*, at steps of 5 s. Values were later averaged at each step to reduce measurement noise.

### A/C_i_ Curves at 2% Oxygen

A gas mixture containing 2% oxygen and 98% nitrogen was fed to the inlet of the LI-6400 XT. Irradiance was set to 1,500 µmol m^−2^ s^−1^, and *C_a_* was set to 150 µbar. After reaching steady-state *A*, *C_a_* was decreased in steps of 130, 100, 70, and 50 µbar. *A* and *C*_i_ were logged as described above. At each *C*_a_, the infrared gas analysers were matched.

### A/C_i_ Curves at 21% Oxygen

Irradiance was set to 1,500 µmol m^−2^ s^−1^, and *C_a_* was set to 400 µbar. After reaching steady-state *A*, *C_a_* was decreased in steps of 300, 200, 130, 100, 70 and 50 µbar. Then, *C_a_* was raised to 400 µbar and after reaching steady-state *A*, *C*_a_ was increased in steps of 600, 750, 900, 1,100, 1,400, 1,700, and 2,000 µbar. *A* and *C_i_* were logged after reaching steady-state (3–5 min per step) as described above. At each *C_a_*, the infrared gas analysers were matched. Parameters describing maximum rate of carboxylation (*V_cmax_*), rate of linear electron transport at the measuring irradiance (*J_1500_*) and triose phosphate utilization capacity (*TPU*) were determined using the excel solver tool by Sharkey (2016). Additionally, operating and maximal fluorescence in light-adapted leaves (*F* and *F_m_*′, respectively) were determined at each *C_a_* by using a multi-phase flash protocol (MPF; Loriaux et al., 2013). The maximum intensity of the MPF was ~9.000 µmol m^−2^ s^−1^, the durations of the three phases were 0.3, 0.7 and 0.4 s respectively, and the percentage decrease of flash intensity during phase two was 60%. These MPF settings were found to yield the most accurate results in pilot experiments (data not shown). Photosystem II operating efficiency (*Φ_PSII_*) was calculated as *Φ_PSII_ = (F_m_*′ − *F)/F_m_*′. Mesophyll conductance (*g_m_*) was determined following the variable *J* method proposed by Harley et al. (1992); the variables *A*, *C_i_*, and *Φ*_PSII_ to calculate *g_m_* were determined at a *C_a_* of 400 µbar and an irradiance of 1500 µmol m^−2^ s^−1^. Parameters to calculate *g_m_*, namely *R_d_*, Γ* and *s*, were determined from *A*/*C_i_* and *A*/PAR measurements at 2 and 21% oxygen following Yin et al. (2009).

### Photosynthetic Induction

Leaves were adapted to 50 µmol m^−2^ s^−1^ until *g_s_* was constant (40–60 minutes). Irradiance was then increased to 1500 µmol m^−2^ s^−1^ in a step change, and gas exchange values were logged every 2 s for 60 min. These measurements were performed at *C_a_* and air humidity close to the plant’s growth conditions (400 µbar *C_a_*, 7 mbar VPD), termed “control” hereafter. Photosynthetic induction was additionally assessed under two other conditions: “high VPD” (15 mbar) and “low CO_2_” (200 µbar), keeping all other conditions the same. During photosynthetic induction, chlorophyll fluorescence was measured using a saturating MPF (described above) once every minute during the first ten minutes, and once every two minutes thereafter. Photosynthetic induction (PI, %) was calculated as a percentage of the total change between initial *A* (*A*_i_) and final *A* (*A_f_*) of each transient: PI = (A-A_i_)/(A_f_-A_i_)*100. Intrinsic water use efficiency (*WUE_i_*) was calculated as *WUE_i_* = *A*/*g_s_*. Non-photochemical quenching (*NPQ*) during photosynthetic induction was calculated as *NPQ = (F_m_* − *F_m_′)/F_m_′*. The coefficient of photochemical quenching (*qP*) and the efficiency of open photosystem II traps (*F_v_′/F_m_′*) were calculated after Oxborough and Baker (1997), as *qP* = *(F_m_′* − *F)/(F_m_′* − *F_o_′)* and *F_v_′/F_m_′* = *(F_m_′* − *F_o_′)/F_m_′*, where *F_o_′* is minimal fluorescence from irradiance- adapted leaves. *F_o_′* was calculated after Oxborough and Baker (1997).

### Statistical Analysis

All statistical tests were performed at *P*=0.05 as threshold for significance. Where appropriate, a two-sided Student’s *t*-test was used to determine significant differences between genotypes. For photosynthetic induction under different environmental conditions, a two-way analysis of variance (ANOVA) was performed, and interaction means were separated based on Fisher’s least significant difference test. Residuals were tested for normal distribution (Shapiro-Wilk test) and equal variances were assumed for treatment groups. If the requirement for normal distribution was not fulfilled, the procedure was repeated on log-transformed data. If after log transformation residuals still did not show normality, Kruskal-Wallis one-way ANOVA, considering six treatments (three environmental conditions times two genotypes), was performed using the original data. In case of significant treatment effects, Dunn’s test of multiple comparisons was performed to identify differences between the six treatments. All statistical tests were performed in Genstat (VSN international, Hempstead, UK) except for Dunn’s test, which was performed in R (R Core Team, 2020) using the dunn.test package (Dinno, 2017).

## Results

### Steady-State CO_2_ and Irradiance Responses of Photosynthesis

**Figure 1:**
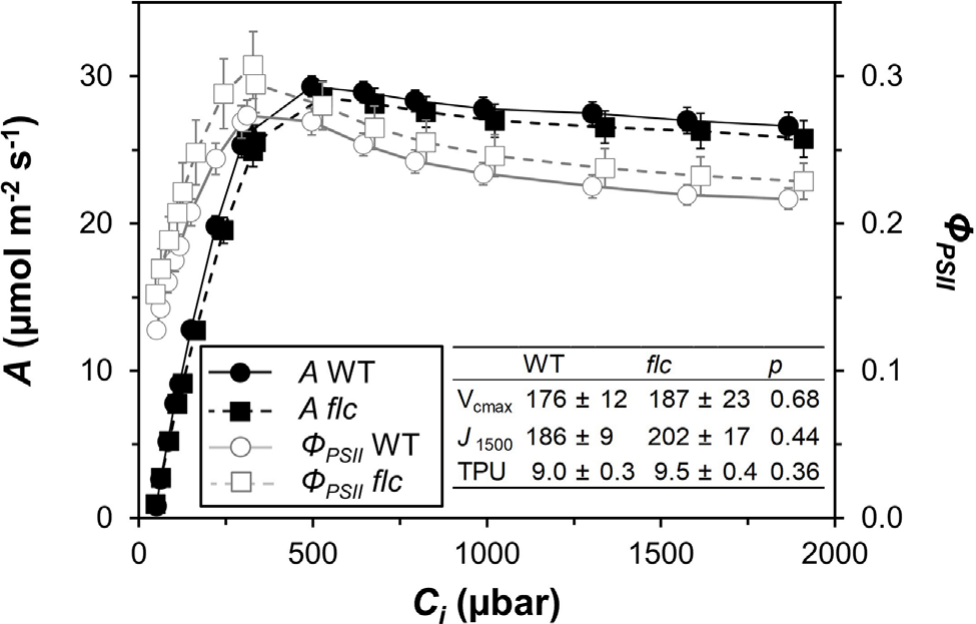
Steady-state responses of net photosynthesis rate (*A*) and photosystem II operating efficiency (*Φ_PSII_*) to leaf internal CO_2_ partial pressure (C_i_), in wildtype (WT) and *flacca* (*flc*) leaves, measured at 21% oxygen. Insert: parameters derived from curve fitting (after Sharkey, 2016) to *A*/C_i_ response (unit for all: μmol m^−2^ s^−1^), namely maximum rate of carboxylation (*V*_cmax_), rate of linear electron transport at 1500 μmol m^−2^ s^−1^ PAR (*J_1500_*) and triose phosphate utilisation capacity (*TPU*); *P* values for comparisons between WT and *flc* based on two-sided t-test. Symbols and numbers represent averages ± SEM, n = 5.

**Figure 2:**
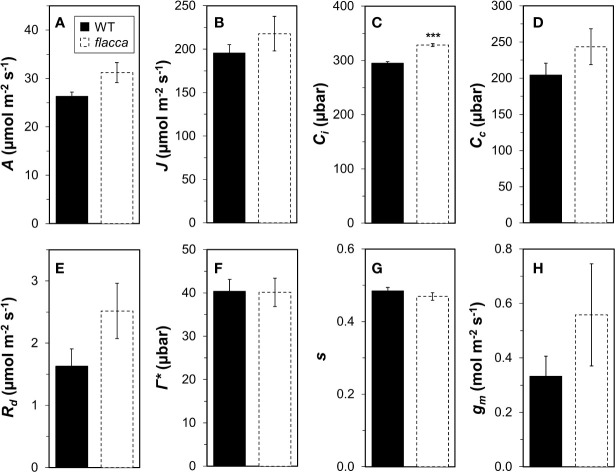
Analysis of mesophyll conductance (*g_m_*) and its components in wildtype (WT) and *flacca* leaves. **(A)** net photosynthesis rate (*A*); **(B)**, linear electron transport rate (*J*); **(C)** substomatal CO_2_ partial pressure (C_i_); **(D)** chloroplast CO_2_ partial pressure (C_c_); **(E)** mitochondrial respiration (*R_d_*); **(F)** CO_2_ compensation point in the absence of mitochondrial respiration (Γ*); **(G)** lumped parameter to convert *Φ_PSII_* to *J* (*s*); **(H)**, *g_m_*. *A*, *J* and *C_i_* were determined under 1000 μmol m^−2^ s^−1^ PAR, 400 μbar CO_2_ atmospheric partial pressure and 25°C chamber temperature. *R_d_*, Γ*, and *s* were determined from *A*/*C_i_* and *A*/PAR curves under photorespiratory and non-photorespiratory conditions after Yin et al. (2009). Bars and error bars represent averages ± SEM, n = 5. ***P<0.01.

Wildtype and *flacca* leaves showed very similar responses of *A* and *Φ_PSII_* to *C_i_* (**Figure 1**). In the CO_2_ range 50 to 300 µbar, *A* increased near-linearly, then peaked at ~500 µbar and with further increases in *C_i_*, *A* declined in both genotypes. Compared to *A*, *Φ_PSII_* peaked at lower *C_i_* (~300 µbar) and exhibited a stronger decline with further increases in *C_i_*. Parameters describing photosynthetic capacity, i.e., *V_cmax_*, *J_1500_* and *TPU*, were not significantly different between genotypes (**Figure 1**, insert). Mesophyll conductance and its components were not significantly different between genotypes (except *C_i_*, which was significantly greater in *flacca*, **Figure 2C**), although *flacca* tended to show greater values for *A*, *J*, *R*_d_, and *C_c_* (**Figure 2**). In dark-adapted leaves, *A* was −1.2 ± 0.1 µmol m^−2^ s^−1^ in wildtype and −1.9 ± 0.1 µmol m^−2^ s^−1^ in *flacca* leaves (*p*=0.008). At low irradiance (50 µmol m^−2^ s^−1^), on the other hand, *A* was similar between genotypes (**Table 1**).

**Table 1:** Steady-state gas exchange traits at 50 and 1500 µmol m^−2^ s^−1^ PAR.

Treatment	Gen	50 µmol m^−2^ s^−1^												1500 µmol m^−2^ s^−1^											
		*A*				*g_s_*				*C_i_*				*A*				*g_s_*				*C_i_*			
Control	WT	2.0	±	0.1	b	0.22	±	0.04	b	379	±	4	c	26.0	±	1.0	B	0.60	±	0.04	b	311	±	2	bc
	*flc*	1.9	±	0.1	b	0.62	±	0.06	d	390	±	1	d	28.4	±	1.4	B	1.03	±	0.05	de	336	±	3	c
Low CO_2_	WT	1.7	±	0.1	a	0.34	±	0.04	c	192	±	1	a	14.8	±	0.6	A	0.71	±	0.04	c	159	±	1	a
	*flc*	1.7	±	0.1	a	0.58	±	0.09	d	194	±	1	ab	16.7	±	1.2	Ab	1.09	±	0.06	e	165	±	1	a
High VPD	WT	1.9	±	0.1	ab	0.14	±	0.01	a	369	±	2	bc	24.4	±	0.8	B	0.41	±	0.01	a	281	±	3	ab
	*flc*	1.8	±	0.1	ab	0.58	±	0.07	d	385	±	1	cd	26.6	±	1.5	B	0.88	±	0.06	d	329	±	2	bc
Genotype																									
Treatment					*																				
Treatment x Gen									*												*				
Kruskal-Wallis χ^2^													***				***								***

Wildtype and flacca leaves were exposed to 400 µbar CO_2_ and 7 mbar VPD (Control), 200 instead of 400 µbar (“low CO_2_”) and 15 instead of 7 mbar (“high VPD”). Net photosynthesis rate (A, µmol m^−2^ s^−1^), stomatal conductance (g_s_, mol m^−2^ s^−1^) and leaf internal CO_2_ partial pressure (C_i_, µbar) are shown as averages ± SEM. Two-way ANOVA was used to indicate significant effects of genotype, treatment or genotype x treatment interactions. When appropriate, a Kruskal-Wallis one-way ANOVA was used. Symbols: *P<0.05, ***P<0.001. Different letters per column indicate statistically significant (P=0.05) differences between treatments as determined by Fisher’s least significant difference test or by Dunn’s test (in case of Kruskal-Wallis one-way ANOVA).

### Response of Photosynthetic Gas Exchange to a Stepwise Irradiance Increase

**Figure 3:**
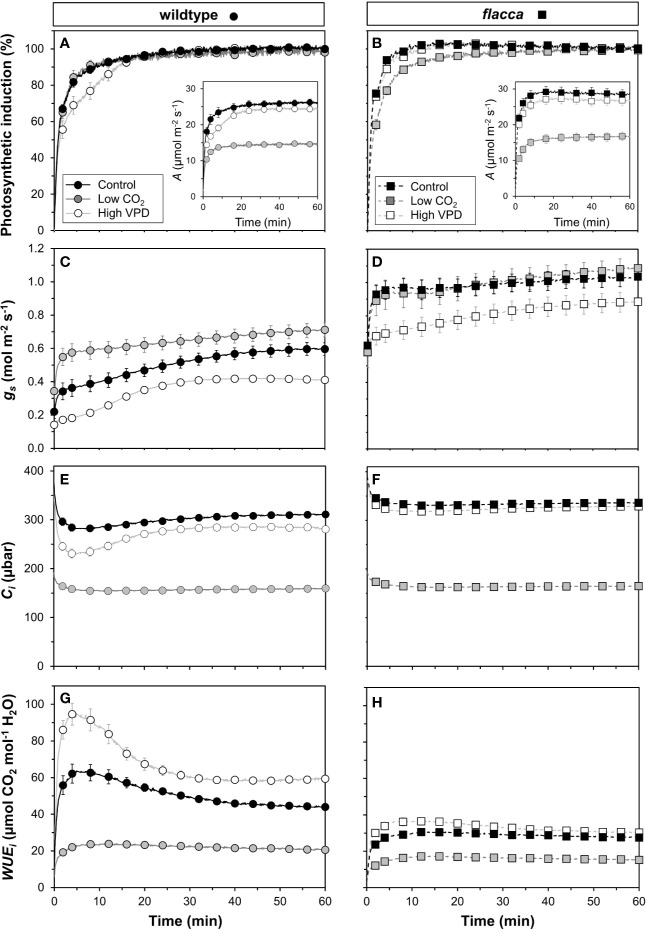
Time courses of gas exchange in wildtype (left panel) and *flacca* leaves (right panel) after a transition from low to high irradiance: **(A, B)** photosynthetic induction; **(C, D)** stomatal conductance (*g_s_*); **(E, F)** leaf internal CO_2_ partial pressure (*C_i_*); **(G, H)** intrinsic water use efficiency (*WUE_i_*). Insets in **(A, B)** show net photosynthesis rates (*A*). Leaves initially adapted to 50 µmol m^−2^ s^−1^ PAR were exposed to 1500 µmol m^−2^ s^−1^ PAR at time = 0 min. Data were logged at 400 µbar CO_2_ and 7 mbar leaf-to-air VPD (Control), 200 instead of 400 µbar (Low CO_2_) and 15 instead of 7 mbar (High VPD). Lines and symbols represent averages ± SEM, n = 5.

**Figure 4:**
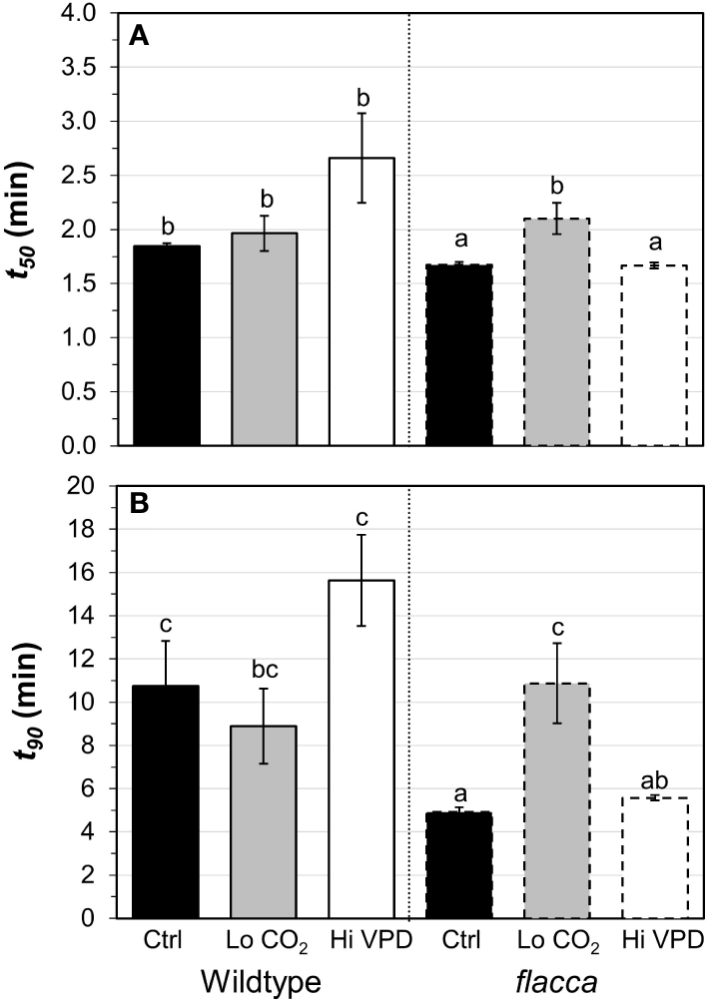
Time to reach 50 (**A**, *t_50_*) and 90% (**B**, *t_90_*) full photosynthetic induction of wildtype and *flacca* leaves after a step change from low to high irradiance. Experimental conditions identical to those described under **Figure 3**. Different letters depict significant differences (*P*<0.05) between ranks, as determined by Dunn’s test. Bars and error bars represent averages ± SEM, n = 5.

Next, we tested how gas exchange in wildtype and *flacca* leaves that had been adapted to low irradiance (50 µmol m^−2^ s^−1^) reacted to a stepwise increase to high irradiance (1500 µmol m^−2^ s^−1^). In wildtype leaves, the rate of photosynthetic induction was slower at high VPD, compared to the other two treatments (low CO_2_ or high VPD; **Figure 3A**), while in *flacca*, there was no difference between control and high VPD treatments (**Figure 3B**). However, while in wildtype leaves the rate of photosynthetic induction was the same in the control and low CO_2_ treatments, in *flacca*, induction at low CO_2_ was slower than in the control treatment (**Figures 3A, B**). The *flacca* mutation had significant effects on the times to reach 50 (*t_50_*) and 90% (*t_90_*) of full photosynthetic induction: under control conditions, *t_50_* was 91% and *t_90_* was 46% of wildtype values in *flacca*, while under high VPD, *t_50_* was 63% and *t_90_* was 36% of wildtype values in *flacca* (**Figure 4**). Both indices were not significantly different between genotypes under low CO_2_ (**Figure 4**). Transient *A* was higher in *flacca* than in wildtype leaves, and in both genotypes was slightly higher in control than in high VPD, as well as substantially reduced at low CO_2_ (insets in **Figures 3A, B**; **Table 1**).

In *flacca*, *A* showed a small decrease between ~1.5 and 2.0 min after the stepwise increase in irradiance under control and high VPD conditions (**Figure S1B**). These dynamics are very similar to those previously seen in shade-adapted wildtype tomato leaves undergoing photosynthetic induction under high CO_2_ partial pressure (Kaiser et al., 2017b), and in the present study were observed neither at low CO_2_ (**Figure S1B**), nor in wildtype leaves (**Figure S1A**). The most likely explanation for this phenomenon is a transient mismatch between the rate of CO_2_ fixation in the Calvin cycle and downstream sucrose metabolism, which would transiently limit the availability of free phosphate in the chloroplast (Prinsley and Leegood, 1986; Stitt and Grosse, 1988; Stitt and Quick, 1989).

During the complete trajectory of photosynthetic induction, stomatal conductance (*g_s_*) in wildtype leaves was strongly increased by lowering CO_2_, and substantially reduced by increasing VPD (**Figure 3C**; **Table 1**). In *flacca*, *g_s_* was lower in the high VPD treatment compared to both other treatments during photosynthetic induction (**Figure 3D**; **Table 1**). Intriguingly, while the low irradiance adapted (initial) *g_s_* in wildtype leaves reacted to the treatment levels in a predictable way, i.e., decreasing under high VPD and increasing under low CO_2_ relative to control (**Figure 3C**), initial *g_s_* in *flacca* did not (**Figure 3D**; **Table 1**). Also, *g_s_* in *flacca* was substantially higher than that in wildtype leaves in all cases, as would be expected of an ABA mutant.

In control conditions, *C_i_* in wildtype leaves showed an initial decrease in the first 10 minutes of photosynthetic induction, after which it gradually increased as stomata opened (**Figure 3E**). This decrease was exacerbated in the high VPD treatment. Even after partial recovery of *C_i_* due to stomatal opening, *C_i_* did not reach control values when approaching steady state (**Figure 3E**; **Table 1**). In *flacca* leaves, *C_i_* decreased less strongly under both control and high VPD conditions, and did not increase much during the remainder of photosynthetic induction (**Figure 3F**). Also, *C_i_* time courses in both of these treatments were virtually indistinguishable in *flacca*, which is explained by the diminished reduction of *g_s_* under high VPD (**Figure 3D**). Under low CO_2_, *C_i_* was similar in wildtype and *flacca*, displaying only small decreases in the beginning of photosynthetic induction without subsequent recovery (**Figures 3E, F**).

Intrinsic water use efficiency (*WUE_i_*) was roughly twice as high in wildtype compared to *flacca* leaves (**Figures 3G, H**). In the wildtype, a high VPD resulted in large increases (+31%), and a low CO_2_ in large decreases (−56%), of *WUE_i_* relative to control conditions (**Figure 3G**). In *flacca*, *WUE_i_* was markedly reduced (−50%) under low CO_2_ relative to the other two conditions (**Figure 3H**). While *WUE_i_* showed strong dynamics in the first 30 minutes after exposure to high irradiance under high VPD and control conditions in the wildtype, it plateaued early after an initial increase (<5 min) in all other cases (**Figures 3G, H**).

### Chlorophyll Fluorescence Dynamics During Photosynthetic Induction

**Figure 5:**
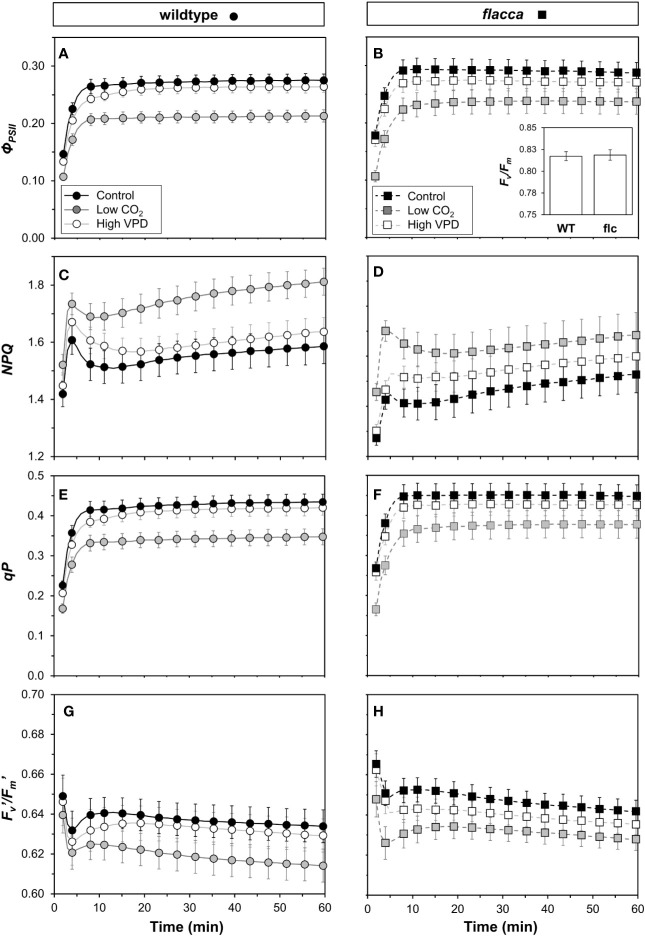
Time courses of chlorophyll fluorescence in wildtype (WT, left panel) and *flacca* leaves (*flc*, right panel) after a transition from low to high irradiance. **(A, B)** photosystem II operating efficiency (*Φ_PSII_*); **(C, D)** non-photochemical chlorophyll fluorescence quenching (*NPQ*); **(E, F)** coefficient of photochemical quenching (*qP*); **(G, H)** efficiency of open photosystem II traps (*F_v_*′*/F_m_*′). Experimental conditions identical to those described under **Figure 3**. Lines and symbols represent averages ± SEM, n = 5.

In wildtype leaves, an initial rapid increase in *Φ_PSII_* within the first ~8 min was followed by a slower, more gradual increase towards a steady state under control and high VPD conditions until ~40 min (**Figure 5A**). In *flacca* leaves in control and high VPD conditions, a gradual decrease was observed after the initial increase in *Φ_PSII_* (**Figure 5B**). In both genotypes, *Φ_PSII_* under low CO_2_ stabilized quickly at lower values and then plateaued. Dark-adapted *F_v_/F_m_* was ~0.82 in both genotypes (n.s., **Figure 5B**, inset). A rapid increase in *NPQ* in the first five minutes was followed by a decrease until 10 to 20 min, which was followed by a slower increase until the final measurement after 60 minutes (**Figures 5C, D**). Under control and high VPD conditions, *NPQ* initially rose to much higher values in wildtype (1.6–1.7) compared to *flacca* leaves (1.4–1.5). The subsequent decrease to a local minimum showed a greater amplitude in wildtype (~0.1) compared to *flacca* leaves (~0.05). Under low CO_2_, *NPQ* tended to be greater in both genotypes compared to the other treatments. The coefficient of photochemical quenching (*qP*) showed dynamics similar to those of *Φ_PSII_* (**Figures 5E, F**). *qP* and *Φ_PSII_* were highly correlated in all treatments (R^2^ >0.99). The efficiency of open photosystem II traps (*F_v_*′*/F_m_*′) showed dynamics that were the inverse of those of *NPQ* (**Figures 5G, H**); *NPQ* and *F_v_*′*/F_m_*′ were highly correlated (R^2^ >0.99). These correlations suggest that *Φ_PSII_* dynamics were largely due to changes in *qP* rather than changes in *F_v_*′*/F_m_*′ or *NPQ* (Baker et al., 2007).

### Stomatal Effects on Rate of Photosynthetic Induction

**Figure 6:**
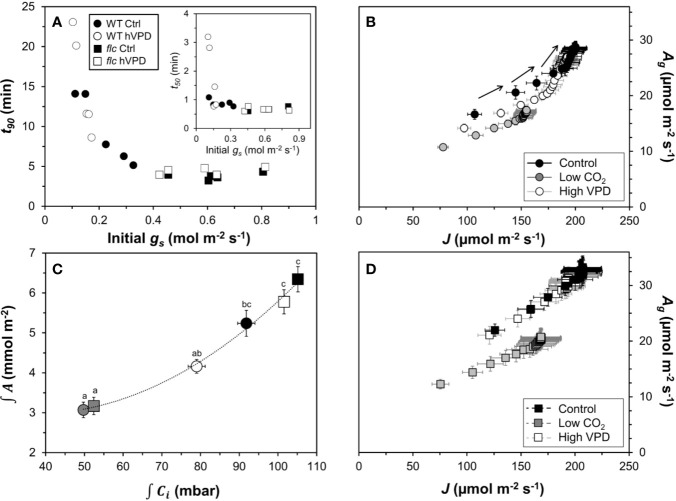
Effects of stomatal conductance on photosynthetic induction. **(A)** relationship between stomatal conductance of leaves adapted to 50 µmol m^−2^ s^−1^ PAR (initial *g_s_*) and time to reach 90% of full photosynthetic induction (*t_90_*) after a switch to high irradiance, inset: relationship between initial *g_s_* and *t_50_*; **(B)** relationship between electron transport rate (*J*) and gross photosynthesis rate (*A*_g_) in the wildtype, arrows show sequence in which data were logged; **(C)** relationship between integrated net photosynthesis rate (∫*A*) and integrated leaf internal CO_2_ partial pressure (∫*C_i_*) during the first five minutes of photosynthetic induction, different letters indicate statistically significant (*P*<0.05) differences between treatments; **(D)** relationship between *J* and *A*_g_ in *flacca*. Experimental conditions identical to those described under **Figure 3**. Symbols represent averages ± SEM, n = 5.

Next, we explored several ways to visualize the effects of (partially) closed stomata on photosynthetic induction. First, initial, low-irradiance adapted *g_s_* was plotted against *t_90_* (**Figure 6A**). Across both genotypes, there was a consistent threshold-type relationship between initial *g_s_* and *t_90_*: at initial *g_s_* <0.4 mol m^−2^ s^−1^, *t_90_* increased strongly with decreases in initial *g_s_*, reaching values of ~15 min at an initial *g_s_* of 0.11 mol m^−2^ s^−1^. At initial *g_s_* >0.4 mol m^−2^ s^−1^, the value of *t_90_* (~5 min) was unaffected by further increases in initial *g_s_*. Roughly, a similar threshold was visible between *t_50_* and initial *g_s_*, as initial *g_s_* <0.2 mol m^−2^ s^−1^ tended to increase *t_50_*, while *t_50_* was unaffected by differences in initial *g_s_* in the range 0.2 to 0.8 (**Figure 6A**, inset). Initial *g_s_* versus *t_50_* or *t_90_* did not show a similar relationship at low CO_2_ (**Figure S2**) and was therefore omitted from **Figure 6A**.

Photosynthesis integrated over the initial five minutes of photosynthetic induction scaled well with *C_i_* integrated over the same period (**Figure 6C**). This suggests that *A* was affected by *C_i_*, while the change in *C_i_* was due to treatment and/or genotype effects on *g_s_*. Finally, plotting *J* vs. gross photosynthesis rates (*A*_g_, calculated as *A* plus *R_d_*) produced very similar results between control and high VPD conditions in *flacca* leaves (**Figure 6D**), while in wildtype leaves *A*_g_ showed higher values for the same *J* in control compared to high VPD conditions (**Figure 6B**). Under low CO_2_, both genotypes showed decreased *A*_g_ for a given *J*. Also, while in *flacca* leaves the plots of *A*_g_ vs. *J* were highly linear (**Figure 6D**), in wildtype leaves they showed an upwards curvature at higher *A*_g_/*J* values, i.e., *A*_g_ increased more strongly than *J* (**Figure 6B**). This upwards curvature is indicative of an increase in the rate of carboxylation relative to the rate of oxygenation, most likely caused by an increase in *C_i_* due to increased stomatal opening (Kaiser et al., 2017a; Zhang et al., 2018).

## Discussion

In recent years, the dynamic responses of photosynthesis to fluctuating light, and their limitations, have received more attention. It is now widely recognised that increasing photosynthesis is a pathway to increasing crop productivity (Ort et al., 2015), that crops frequently encounter light intensity fluctuations (Kaiser et al., 2018), and that alleviating some of the limitations acting on photosynthesis transients can strongly increase biomass (Kromdijk et al., 2016). Indeed, research looking into reductions of limitations acting on dynamic photosynthesis currently receives a lot of attention (Tanaka et al., 2019; Acevedo-Siaca et al., 2020; Kimura et al., 2020; Yamori et al., 2020).

### Greater Stomatal Conductance Increases the Rate of Photosynthetic Induction

Responses of *A* to *C_i_*, as well as mesophyll conductance, were similar between the *flacca* mutant and the wildtype (**Figures 1** and **2**), while stomatal conductance was strongly enhanced in *flacca* leaves (**Figures 3C, D**). This confirms our hypothesis that the *flacca* mutant is a useful system for strongly reducing stomatal limitations and, via their reduction, better understanding their effects. This is similar to earlier reports using genotypes with “always-open” stomata (Tomimatsu and Tang, 2012; Tomimatsu et al., 2019; Kimura et al., 2020; Yamori et al., 2020). Our results further suggest that the faster photosynthetic induction observed in *flacca* compared to wildtype leaves under control and high VPD conditions (**Figures 3A, B** and **4**) was indeed due to much higher stomatal conductance (difference between genotypes in initial *g_s_* was ~0.4 mol m^−2^ s^−1^). Initial *g_s_* in leaves adapted to darkness or shade strongly impacts on rates of photosynthetic induction upon illumination with high irradiance. It has been reported previously that parameters such as the time needed to reach 50 or 90% of full photosynthetic induction (*t_50_* and *t_90_*, respectively) show a strong bimodal relationship with initial *g_s_* (Valladares et al., 1997; Allen and Pearcy, 2000; Kaiser et al., 2016), similar as shown in the present study (**Figure 6A**).

The decrease in initial *g_s_* in the wildtype upon high VPD (**Figure 3C**) translated into a marked decrease in *C_i_* during photosynthetic induction (**Figures 3E** and **6C**). This decrease was less strong in control conditions and was barely visible in *flacca* under control or high VPD (**Figure 3F**), as initial *g_s_* in *flacca* did not react to high VPD (**Figure 3D**). The reduced transient availability of *C_i_* decreased the rate of photosynthetic induction in the wildtype under high VPD (**Figure 3A**). This reduction, in turn, fed back on dynamic *Φ_PSII_* (which was slowed down; first 15 minutes in **Figure 5A**), *NPQ* (which initially increased to higher levels and then relaxed less quickly than under control conditions; **Figure 5C**) and the relationship between gross photosynthesis and electron transport (**Figure 6B**). Unlike initial *g_s_*, stomatal opening (difference between initial and final *g_s_*) was not different between wildtype and *flacca* leaves under control (~0.4 mol m^−2^ s^−1^) and high VPD conditions (~0.3 mol m^−2^ s^−1^; **Table 1**).

Perhaps surprisingly, photosynthetic induction was not different between genotypes under low CO_2_ partial pressure (**Figures 3** and **4**). This may be explained in two ways: firstly, initial *g_s_* in the wildtype increased, from 0.22 mol m^−2^ s^−1^ at 400 µbar to 0.34 mol m^−2^ s^−1^ at 200 µbar, while that in *flacca* did not (**Table 1**). This *g_s_* increase in wildtype leaves almost halved the difference in initial *g_s_* between genotypes (0.4 → 0.24 mol m^−2^ s^−1^). Secondly, any positive effect that the remaining difference in initial *g_s_* may have had on photosynthetic induction in *flacca* was probably additionally decreased by low CO_2_ availability.

### Initial *g_s_* in Leaves Lacking ABA Does Not React to Low CO_2_ or High VPD

A striking finding of the present study was that while *g_s_* in wildtype leaves (as expected) increased upon reductions in CO_2_ partial pressure (**Figure 3C**, **Table 1**), *g_s_* in *flacca* was virtually unchanged (**Figure 3D**). This confirms that ABA is part of the CO_2_ signalling pathway in stomatal regulation (reviewed in Engineer et al., 2016). Under high VPD (15 mbar), *g_s_* in *flacca* leaves adapted to low irradiance was similar to that at low VPD (7 mbar; **Figure 3D**). In wildtype leaves, stomata again responded as expected, by reducing their aperture under increased VPD (**Figure 3C**). At high irradiance, however, *g_s_* in *flacca* did respond to the increase in VPD, as its value was reduced by ~0.15 mol m^−2^ s^−1^ compared to that at 7 mbar (**Table 1**). While there is ongoing controversy about the role of ABA in stomatal sensing of humidity, Merilo et al. (2018) showed that a number of genotypes that are either ABA deficient or ABA insensitive closed their stomata when exposed to an increase in VPD (at 150–500 µmol m^−2^ s^−1^ PAR). The authors explained this phenomenon (which is at variance with Mcadam et al., 2015; McAdam et al., 2016) as most likely being a hydropassive response, resulting from much higher initial *g_s_* in ABA mutants, and thus a greater drop in humidity in the substomatal cavity (Merilo et al., 2018).

### Limitations of the Study

In this study, we only used one mutant (*flacca*, LA0673) to examine the effects of open stomata on the rate of photosynthetic induction. Ideally, a larger number of mutants, each resulting in differential *g_s_* relative to its wildtype, should be used; this to make sure that the observed effects on photosynthetic induction were truly caused by *g_s_* rather than some other putative effects of the flacca mutation. Secondly, during growth *flacca* plants were regularly sprayed with ABA, following the recommendations of Imber and Tal (1970). It may be that this ABA application triggered unwanted responses in the plants, although based on all results presented here it seems that photosynthesis was fully functional in *flacca*.

## Conclusions and Outlook

The current study suggests that in wildtype leaves, *g_s_* exerts a substantial limitation on non-steady state photosynthesis. The *flacca* mutant can partially overcome this limitation through stomata that remain open in low light, resulting in substantially reduced *t_90_* in ambient CO_2_ partial pressure (*t_90_* was 36–46% in in *flacca* relative to wildtype values). Nevertheless, while *flacca* is a good system for testing (dynamic) stomatal limitation in the laboratory, breeding for a similar stomatal behaviour will not be useful for most crops (except possibly for production in wetland areas), as this improvement of photosynthesis in fluctuating light will come with a significant reduction in water use efficiency and a major fitness disadvantage. A more promising approach may be to improve stomatal responsiveness to light intensity fluctuations, as this can potentially increase both light and water use efficiency under fluctuating light intensities. Recent, promising examples of increased *g_s_* responsiveness are the BLINK1 transformant (Papanatsiou et al., 2019) and the PATROL1 overexpressor (Kimura et al., 2020).

## Data Availability Statement

All datasets presented in this study are included in the article/**Supplementary Material**.

## Author Contributions

EK and AM designed the study with input from all other authors. EK performed measurements and data analysis. EK wrote the manuscript with input from all other authors.

## Conflict of Interest

The authors declare that the research was conducted in the absence of any commercial or financial relationships that could be construed as a potential conflict of interest.

## Abbreviations

*A*, net photosynthesis rate; *A*_g_, gross photosynthesis rate; ABA, abscisic acid; ANOVA, analysis of variance; *C_a_*, leaf external CO_2_ partial pressure; *C_c_*, chloroplast CO_2_ partial pressure; *C_i_*, substomatal CO_2_ partial pressure; *F*, fluorescence emission from leaves under actinic irradiance; *F_m_*, maximal fluorescence in dark-adapted leaves; *F_m_*’, maximal fluorescence in light-adapted leaves; *F_o_*, minimal fluorescence in dark-adapted leaves; *F_v_/F_m_*, maximum quantum efficiency of PSII photochemistry; *F_v_*′*/F_m_*′, efficiency of open PSII traps; *g_m_*, mesophyll conductance; *g_s_*, stomatal conductance; *J*, rate of linear electron transport; MPF, multi-phase flash protocol; PAR, photosynthetically active radiation; *qP*, coefficient of photochemical quenching; *R_d_*, mitochondrial respiration; *s*, lumped parameter used to convert *Φ_PSII_* to *J*; *TPU*, triose phosphate utilization capacity; *WUE_i_*, intrinsic water use efficiency; *V_cmax_*, maximum rate of carboxylation; VPD, leaf-to-air vapour pressure deficit; *Γ**, CO_2_ compensation point in the absence of mitochondrial respiration; *Φ_PSII_*, photosystem II operating efficiency.
