# Supplementary material for: High Stomatal Conductance in the Tomato Flacca Mutant Allows for Faster Photosynthetic Induction
Source: Front Plant Sci. 2020 Aug 25;11:1317. doi: 10.3389/fpls.2020.01317 (PMC7477092; doi:10.3389/fpls.2020.01317)
Supplement: Supplementary file 1 [file DataSheet_1.docx]

**Supporting information for Kaiser et al.:**

**High stomatal conductance in the tomato flacca mutant allows for faster photosynthetic induction**


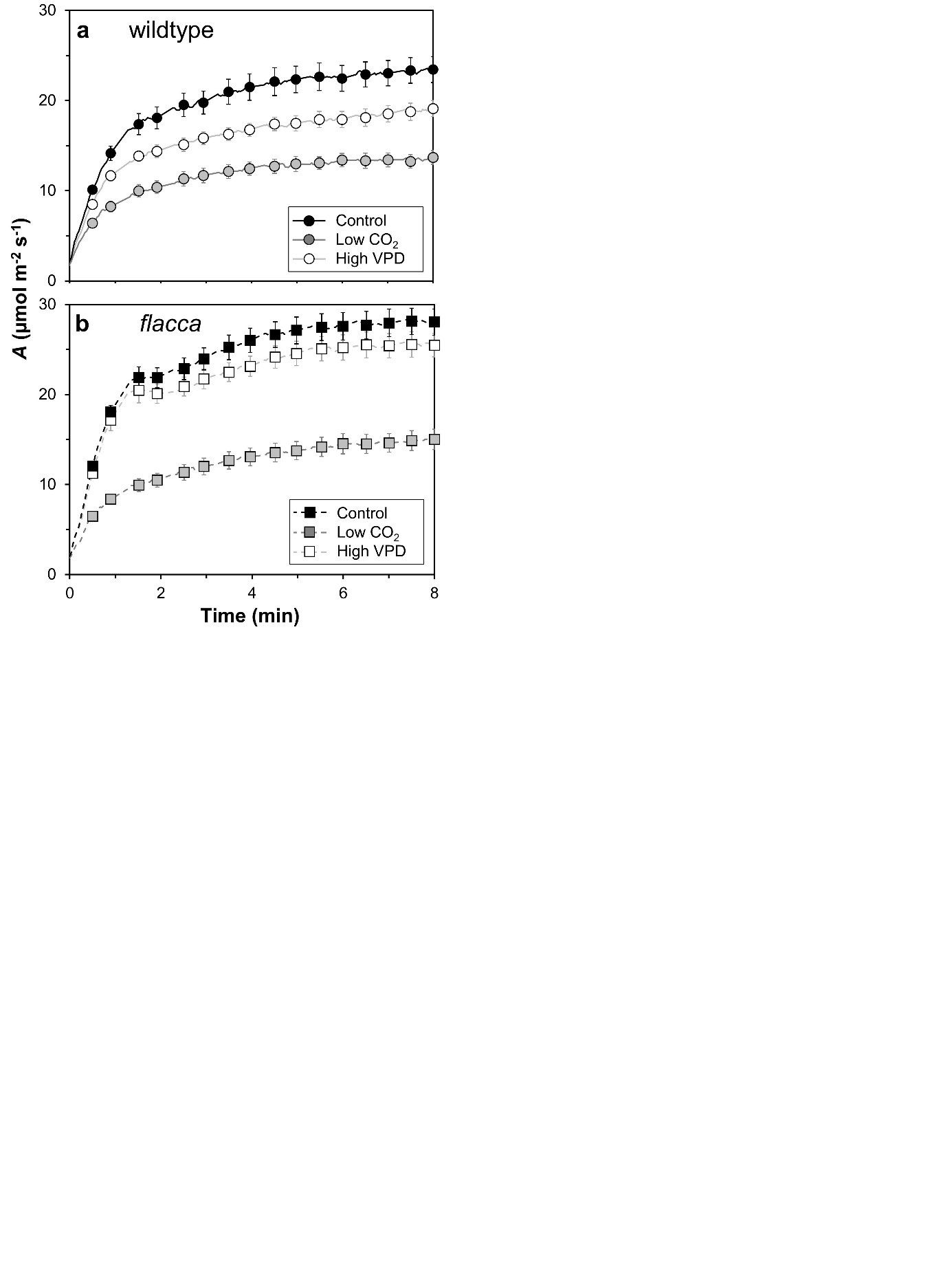


Fig. S1. Time course of net photosynthesis rate (*A*) in the wildtype (a) and *flacca* (b) during the first 8 minutes of photosynthetic induction. Leaves initially adapted to 50 µmol m^-2^ s^-1^ PAR were exposed to 1500 µmol m^-2^ s^-1^ PAR at time = 0 min. Photosynthetic induction was measured at 400 µbar and 0.7 kPa (Control), 200 instead of 400 µbar (Low CO_2_) and 1.5 instead of 0.7 kPa (High VPD). Lines and symbols represent averages ± SEM, n = 5


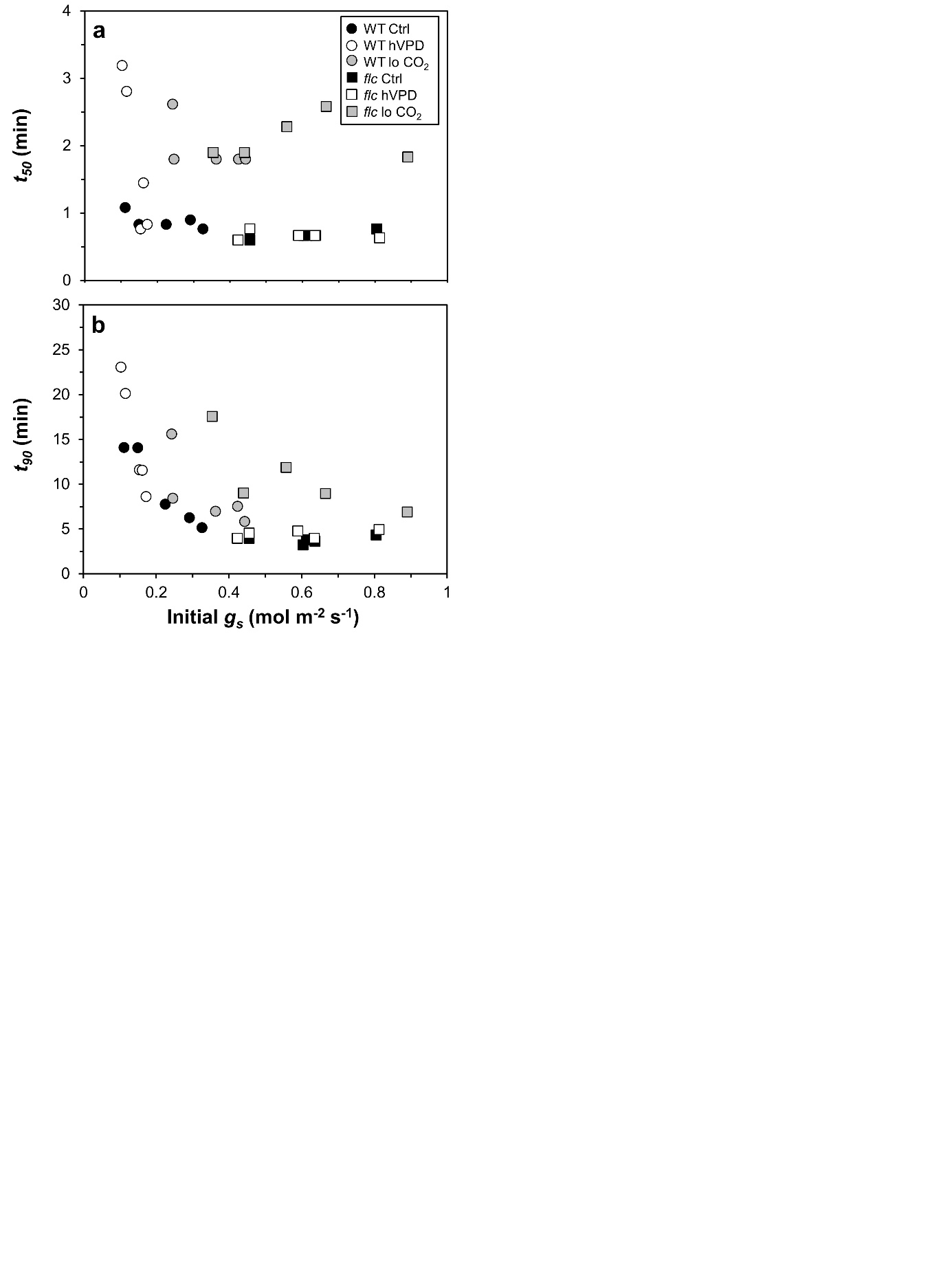


Fig. S2. Relationship between stomatal conductance of leaves adapted to 50 µmol m^-2^ s^-1^ PAR (initial g_s_) and the time required to reach 50% of full photosynthetic induction (a, t_50_), and the time required to reach 90% of full photosynthetic induction (b, t_90_). Data from single wildtype (WT) and flacca (*flc*) leaves undergoing photosynthetic induction at 400 µbar and 0.7 kPa (‘Ctrl’), 200 instead of 400 µbar (‘lo CO_2_’) and 1.5 instead of 0.7 kPa (‘hVPD’) are shown
